# Elastic shell theory for plant cell wall stiffness reveals contributions of cell wall elasticity and turgor pressure in AFM measurement

**DOI:** 10.1038/s41598-022-16880-2

**Published:** 2022-08-01

**Authors:** Satoru Tsugawa, Yuki Yamasaki, Shota Horiguchi, Tianhao Zhang, Takara Muto, Yosuke Nakaso, Kenshiro Ito, Ryu Takebayashi, Kazunori Okano, Eri Akita, Ryohei Yasukuni, Taku Demura, Tetsuro Mimura, Ken’ichi Kawaguchi, Yoichiroh Hosokawa

**Affiliations:** 1 Faculty of Systems Science and Technology, Akita Prefectural University, 84-4 Yurihonjo, Akita, 015-0055 Japan; 2 Division of Materials Science, Nara Institute of Science and Technology, 8916-5 Takayama, Ikoma, Nara, 630-0192 Japan; 3 Institute of Industrial Science, The University of Tokyo, 4-6-1, Komaba, Tokyo, 153-8505 Japan; 4 Yamada Noriaki Structural Design Office Co., Ltd, 1-5-63, Shinagawa, Tokyo, 141-0021 Japan; 5 Graduate School of Engineering, Osaka Institute of Technology, 5-16-1, Ohmiya, Asahi-ku, Osaka, 535-8535 Japan; 6 Division of Biological Science, Nara Institute of Science and Technology, 8916-5 Takayama, Ikoma, Nara, 630-0192 Japan; 7 Department of Biology, Graduate School of Science, Kobe University, 1-1 Rokkodai-cho, Nada-ku, Kobe, 657-8501 Japan; 8 College of Bioscience and Biotechnology, National Cheng-Kung University, Taiwan No.1, University Road, Tainan City, 701 Taiwan

**Keywords:** Biophysics, Computational biophysics

## Abstract

The stiffness of a plant cell in response to an applied force is determined not only by the elasticity of the cell wall but also by turgor pressure and cell geometry, which affect the tension of the cell wall. Although stiffness has been investigated using atomic force microscopy (AFM) and Young’s modulus of the cell wall has occasionally been estimated using the contact-stress theory (Hertz theory), the existence of tension has made the study of stiffness more complex. Elastic shell theory has been proposed as an alternative method; however, the estimation of elasticity remains ambiguous. Here, we used finite element method simulations to verify the formula of the elastic shell theory for onion (*Allium cepa*) cells. We applied the formula and simulations to successfully quantify the turgor pressure and elasticity of a cell in the plane direction using the cell curvature and apparent stiffness measured by AFM. We conclude that tension resulting from turgor pressure regulates cell stiffness, which can be modified by a slight adjustment of turgor pressure in the order of 0.1 MPa. This theoretical analysis reveals a path for understanding forces inherent in plant cells.

## Introduction

An essential factor for understanding the flexibility and diversity of plant morphology is the mechanical properties of cell walls and their response to turgor pressure^1^. Cell wall mechanical properties are determined by many factors such as plant–water relations, the mechanical behavior of materials, and the geometry, shape, and size of the cell^2^. Onion (*Allium cepa*) epidermal cells have been widely used as a model system to investigate mechanical properties because various parameters can be measured experimentally, such as turgor pressure^3–5^, anisotropic cellulose fibrils in the cell wall^6,7^, and the geometry of the epidermal cell surface^8,9^. Cell wall mechanics are associated with cell wall stiffness, referring to the extent to which a material resists deformation in response to an applied force. However, the complex factors mentioned above sometimes make it difficult to interpret the deformation measured by atomic force microscopy (AFM)^8–19^ and relevant indentation tests^20,21^.

**Figure 1:**
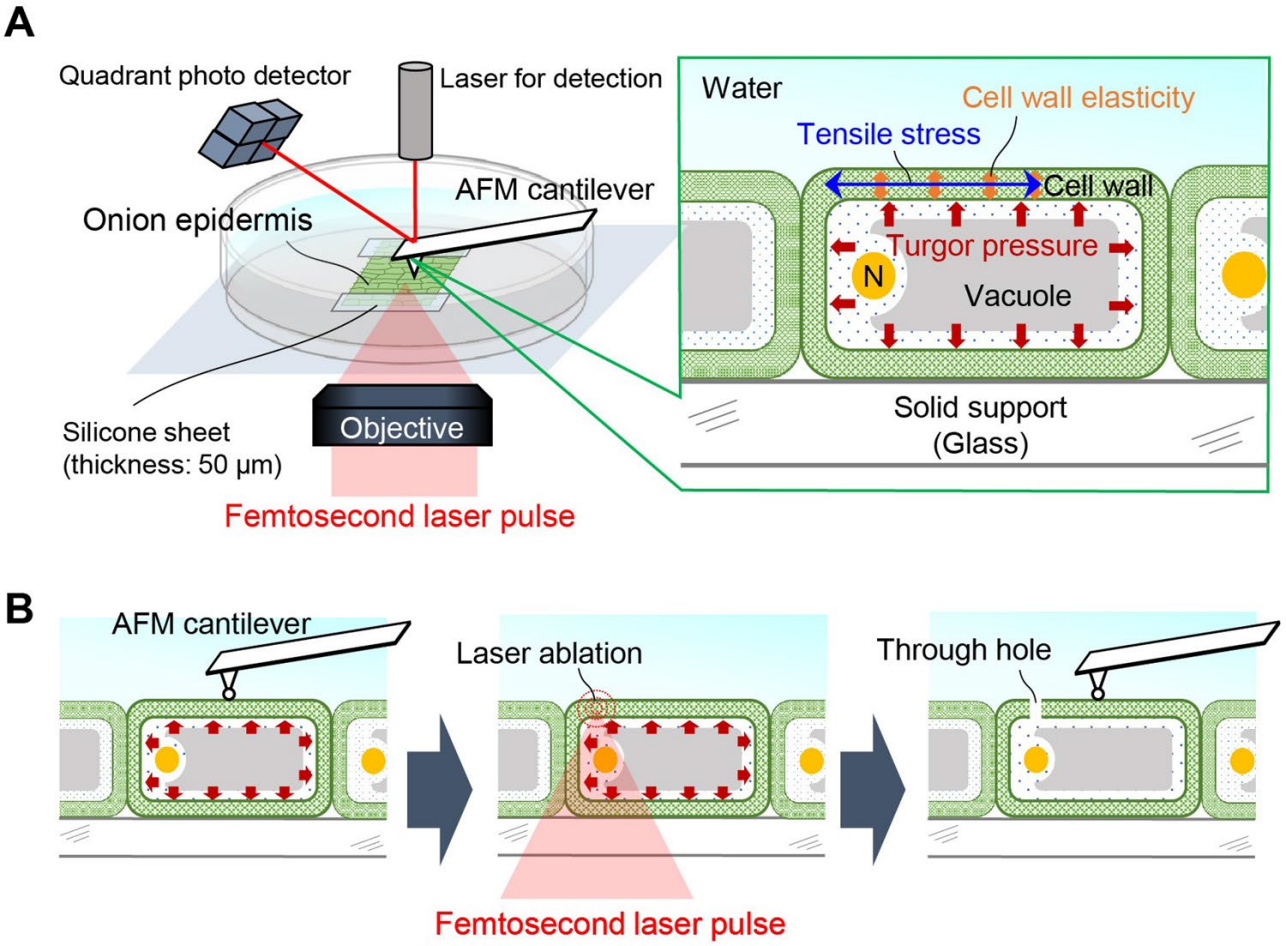
Schematic illustration of laser-assisted AFM measurement of the onion epidermal cell wall. (**A**) Experimental setup of AFM with laser perforation. (**B**) Experimental procedure of AFM detection and perforation using femtosecond laser pulse irradiation to make a through hole.

AFM is a promising method (Fig. 1A) for assessing the so-called apparent stiffness^8–10^, i.e., the slope of the applied force to the indentation depth of the cantilever. Conventionally, measurements of apparent stiffness have been interpreted using the Hertz model^11–17^. In this model, Young’s modulus is estimated based on the assumption of an infinitesimal strain with contact on an elastically homogeneous semi-infinite solid. The Hertz model has been frequently used for evaluating cell wall elasticity; however, especially for plant cells with turgor pressure, some research groups have indicated that deformation of the plant cell wall cannot be described purely by this model when turgor pressure and pre-stress before the indentation are neglected^8–10,18–20^.

**Figure 2:**
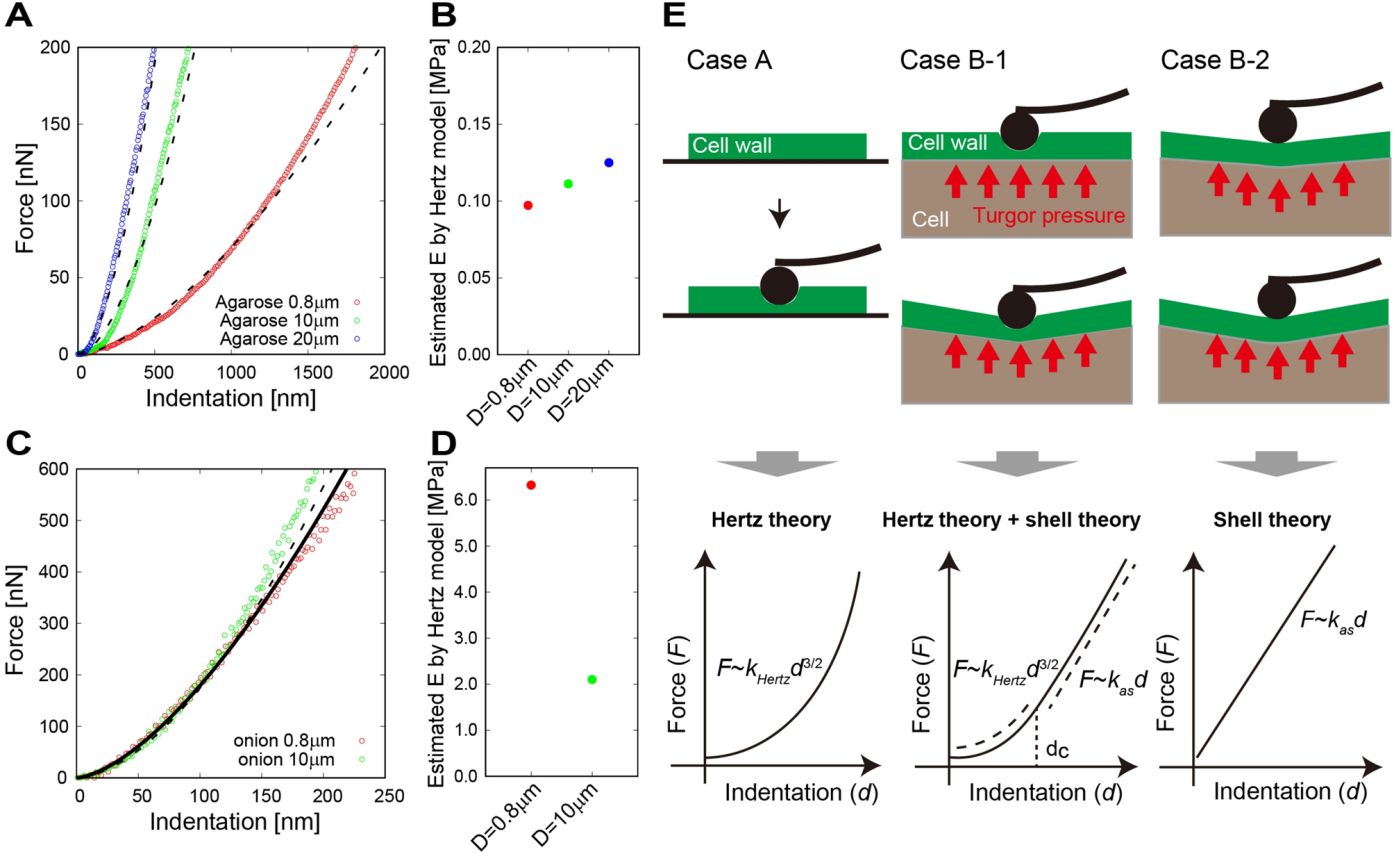
AFM measurement with different cantilever tip size for agarose gel and an onion epidermal cell. (**A**) Force–indentation curve with different tip sizes (0.8, 10, and 20 ﻿µm) for agarose gel. Dashed lines represent curves fitted using the Hertz model. (**B**) Estimated Young’s modulus from the Hertz model. (**C**) Force–indentation curve with different tip sizes (0.8 and 10 ﻿µm) for the onion epidermal cell. Solid line represents the curve for 0.8 ﻿µm, and dashed line represents the curve for 10 ﻿µm fitted using the Hertz model. (**D**) Estimated Young’s modulus from the Hertz model. (**E**) Previously reported interpretations of AFM measurement using a combination of Hertz contact theory^11–16^ and elastic shell theory^9,22–27^. Case A: Fixed cell wall without turgor pressure. In this case, Hertz contact theory can be applied, with force-indentation fitting using *F* ~ *k*_Hertz_*d*^3/2^. Case B: Cell wall under turgor pressure. Scenario B-1 (cantilever indents and warps the cell wall): force-indentation can be fitted usin *F* ~ *k*_Hertz_*d*^3/2^ if *d* < *d*_c_ and* F* ~ *k*_as_*d* if *d* > *d*_c_, where﻿ *d*_c_ can be smaller than the diameter of the cantilever (300 nm in ref. 9). Scenario B-2 (cantilever only warps the cell wall): force-indentation can be fitted using ﻿﻿* F* ~ *k*_as_*d*.

An alternative model is a contact model based on elastic shell theory^9^, in which the cell wall is assumed to be a thin, curved surface pushed by turgor pressure^23–28^ (Fig. 2). This theory enables one to infer turgor pressure from the apparent stiffness in some cases^9,23–31^. In the unified formula from the elastic shell theory^27,28^, AFM indentation is described as the contributions of cell wall elasticity and turgor pressure, while the estimation of elasticity and pressure remains ambiguous. In this study, we further optimized the formula to analyze the apparent stiffness observed from the AFM measurement based on the elastic shell theory. Reliability was verified using finite element method (FEM) simulations. We constructed a simulation model based on experimental results obtained from AFM measurements of an onion epidermal cell. The laser perforation method was applied before AFM measurements to modify turgor pressure. Using the optimized formula, we could quantify not only the turgor pressure but also the cell wall elasticity in the plane direction from the apparent stiffness and cell surface geometry acquired by AFM topography measurements. From these quantitative results, we discuss contributions of these factors to cell wall stiffness.

## Results

### AFM measurements of an onion epidermal cell

To confirm the adaptability of the Hertz model for AFM measurements of plant cells, we compared the force–indentation curve of an onion epidermal cell with that of agarose gel, on which the Hertz model is satisfied, i.e., the force–indentation curves depend on the shape of the cantilever tip, as shown in Fig. 2A. Young’s modulus estimated using the Hertz model (see Methods) was 0.097, 0.111, and 0.125 MPa for the cantilever with tip sizes of 0.8, 10, and 20 µm, respectively (Fig. 2B). Each estimated value was almost same (0.111 on average), with relative error 1%, independent of cantilever tip size. This result indicates that the tip sinks into the material as interpreted by the Hertz contact theory (case A in Fig. 2E).

By contrast, the force–indentation curve for the onion epidermal cell hardly depended on the tip size, as shown in Fig. 2C. When the Hertz model was applied to the curves, the Young’s modulus was estimated to be 6.33 and 2.10 MPa for 0.8 and 10 ﻿µm (Fig. 2D), respectively; these are not similar values compared with the former results (relative error was more than 60%). Namely, the result was hardly explained by the Hertz model alone. The fundamental difference between agarose gel and onion epidermal cells is the inherent structure; the onion cell is a shell-like structure in which the cell wall (shell component) is pushed by turgor pressure, while agarose gel is an elastic semi-infinite solid. Therefore, we considered that the elastic shell model is applicable to the onion epidermal cell in addition to or instead of the Hertz model (cases B-1 or B-2 in Fig. 2E).

**Figure 3:**
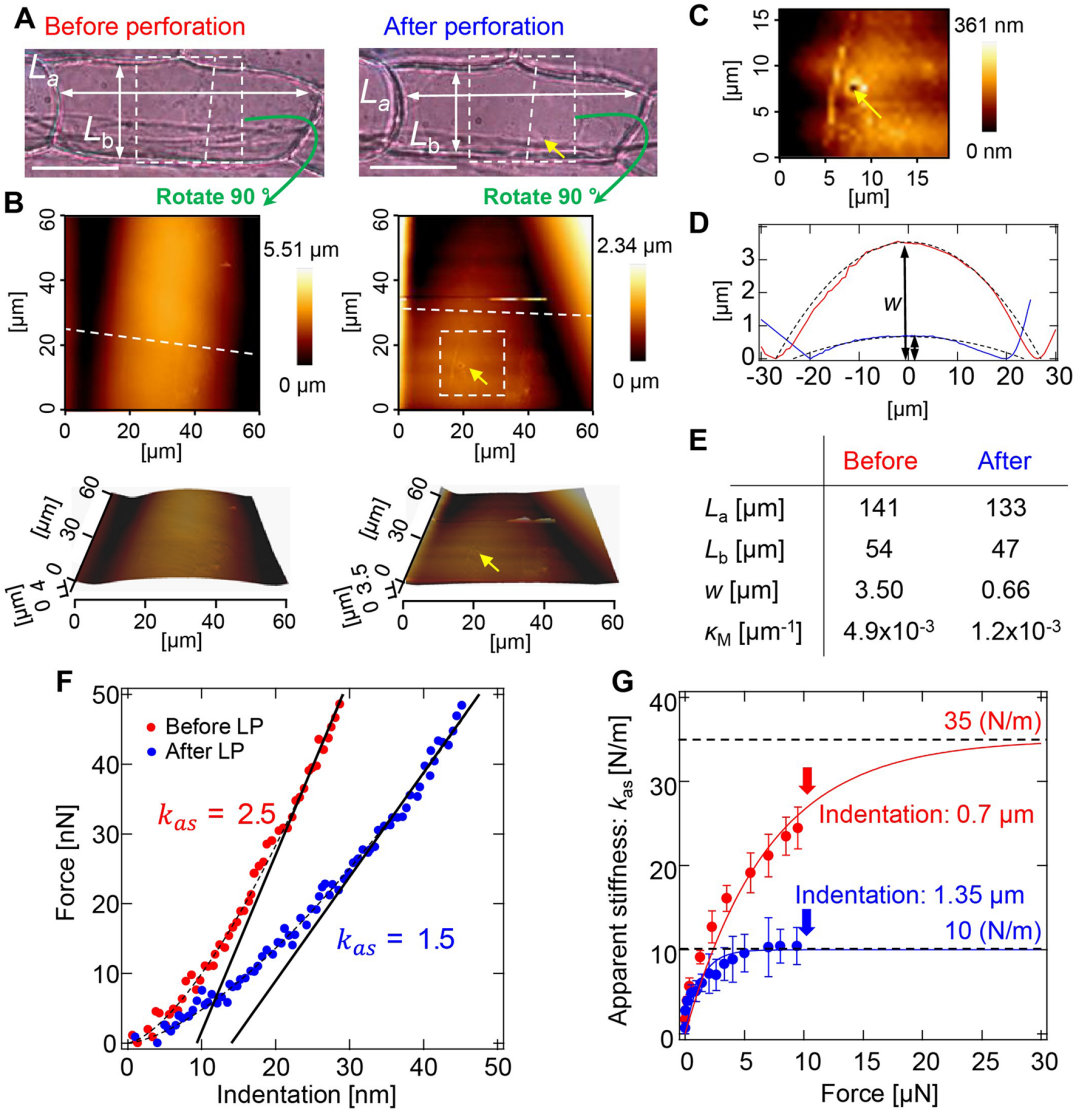
AFM measurement of an onion epidermal cell with laser perforation. (**A**) Photographs of the cell measured before (left) and after (right) perforation. Yellow arrow indicates the perforation point. Cell lengths along long- and short- axes are denoted by *L*_a_ and *L*_b_, respectively. Bars, 50 µm. (**B**) Topographic images before (left) and after (right) perforation. Measurement area corresponds to the dashed box area in (**A**). Lower images are three-dimensional images of upper images. (**C**) Enlarged image of the perforation point. (**D**) Cross-sectional graph of the cell wall surface before (red line) and after (blue line) perforation, corresponding to the height of dashed lines in upper-left and -right images in (**B**), respectively. Bulge height of the cell surface is denoted by *w*. Dashed lines are curves for curvature calculated from *L*_b_ and *w*. (**E**) Quantities determined from AFM measurement. Mean curvature of the cell wall surface *κ*_M_ is calculated from *L*_a_, *L*_b_, and *w*. (**F**) Force–indentation curves of the cell wall before (red dots) and after (blue dots) perforation. Dashed lines are fitting curves by the Hertz model and solid lines are fitting lines by the shell model. (**G**) Apparent stiffness ﻿*k*_as_ as a function of force *F* applied to the cell wall before (red dots) and after (blue dots) perforation. ﻿*k*_as_ is estimated by linear least squares fitting of the force-indentation curve in the vicinity of the *F*, as shown in (F). Bars on dots represent root mean squared error. Solid lines are exponential plateau curves: *k*_as_ = 35 × {1 − exp(− *F*/7)} (red line); *k*_as_ = 10 × {1 − exp(− *F*/1.28)} (blue line).

To obtain geometric parameters of the onion epidermal cell to apply the shell model, we observed the surface structure of the onion epidermal cell before and after laser perforation (LP) (Fig. 1B), whose representative result is shown in Fig. 3. Cell lengths along long- and short- axes (*L*_a_ and *L*_b_, respectively) were measured from photographs (Fig. 3A). Three-dimensional surface geometry of the cell surface was constructed by AFM topography imaging (Fig. 3B). Yellow arrows in the right middle and bottom images indicate the LP point, which is enlarged in Fig. 3C. The LP created a hole with a 2-µm diameter. The cross-sectional graphs in Fig. 3D show shrinkage of cell walls after LP. These observations indicate that the onion epidermal cell surface can be approximated by a cylindrical geometry. The cylindrical shell curvature of the cell wall was decreased after the turgor pressure of the cell was released by LP (﻿*κ*_M_ in Fig. 3E). The force–indentation curve (Fig. 3F) and the apparent stiffness were also modified by LP (Fig. 3G). We have obtained similar results over 5 samples. In addition, the surface geometry and force-indentation curve before LP are comparable with experimental results performed by Beauzamy et al.^9^. Therefore, we used parameters obtained by this experiment as a representative example for the simulation to verify the elastic shell theory. Force–indentation dependence will be discussed later.

### Formulation of apparent stiffness based on elastic shell theory

**Figure 4:**
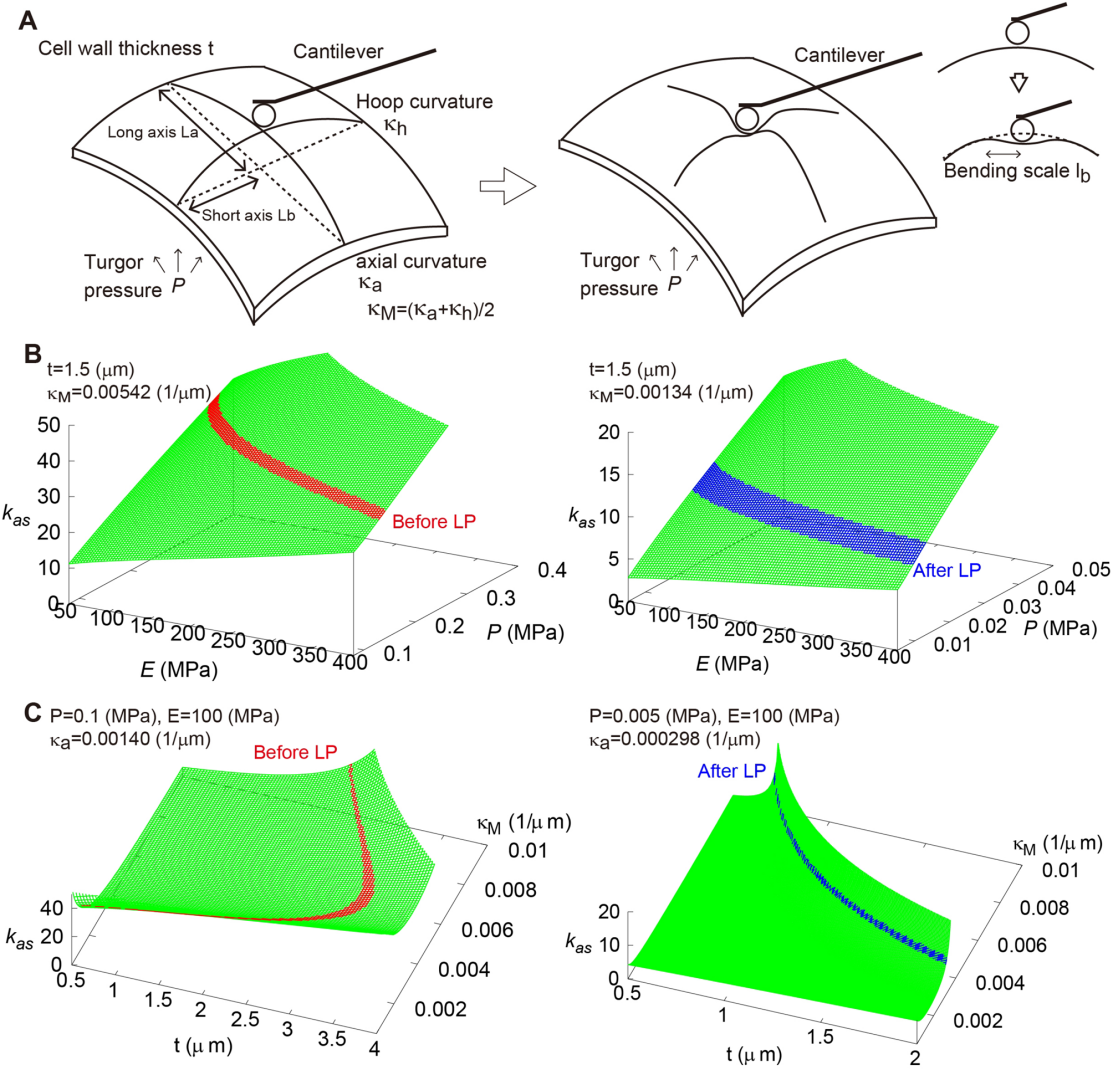
Theoretical evaluation of apparent stiffness of the cell wall and estimation of Young’s modulus and turgor pressure based on AFM measurements. (**A**) Schematic illustrations of shell structure and the indentation process. (**B**) Apparent stiffness﻿ *k*_as_ as functions of *E* and *P* before (left) and after (right) LP. (**C**) Apparent stiffness ﻿*k*_as_ as functions of *t* and ﻿*κ*_M_ before (left) and after (right) LP.

Based on observations of onion epidermal cell geometry, we formulated the apparent stiffness, in which cell wall elasticity as Young’s modulus ﻿*E* and turgor pressure as *P* are taken into consideration. With respect to previous literatures^26,27,30^, the mechanical properties of the onion epidermis during the indentation test were formulated using the elastic shell theory (Fig. 4A). The displacement ﻿*y* perpendicular to the indentation surface in the polar geometry with radial coordinate ﻿*r* is governed by^26^

1 $$B\nabla^{4} y\left( r \right) - \sigma_{\infty } \nabla^{2} y\left( r \right) + Et\kappa_{M}^{2} y\left( r \right) = - \frac{F}{2\pi }\frac{\delta \left( r \right)}{r}.$$

The bending stiffness ﻿*B* includes ﻿*E* (Eq. 8 in Method), the Poisson ratio ﻿*ν* and shell (cell wall) thickness ﻿*t*. *κ*_M_﻿ is the mean curvature of the shell (Eq. 9), and *﻿F* is the force applied by the cantilever. The point force loading is expressed by the Dirac ﻿δ-function. Uniform stress ﻿σ_∞_ on the surface is due to *P*, taking ﻿*κ*_M_ into account (Eq. 10). Using the zeroth order Hankel transformation, the solution of Eq. (1) is

2 $$\begin{aligned} & y\left( r \right) = \frac{{Fl_{b}^{2} }}{{4\pi B\sqrt {\tau^{2} - 1} }}\left( {K_{0} \left( {\frac{{\lambda_{ + }^{\frac{1}{2}} r}}{{l_{b} }}} \right) - K_{0} \left( {\frac{{\lambda_{ - }^{\frac{1}{2}} r}}{{l_{b} }}} \right)} \right) \\ & \lambda_{ \pm } = \tau \pm \left( {\tau^{2} - 1} \right)^{\frac{1}{2}} , \\ \end{aligned}$$

The bending scale ﻿*l*_*b*_ includes *B*, *E*, *t*, and *κ*_M_ (Eq. 14). Dimensionless pressure *τ* includes *P*, *E*, *ν*, *t*,﻿ *κ*_M_, and *κ*_*G*_ (Eq. 15). The function *K*_*0*_(*x*) is the modified Bessel function of zeroth order^32^. From Eq. (2), the apparent stiffness *k*_*as*_ is formulated as

3 $$k_{as} = \mathop {\lim }\limits_{r \to 0} \frac{F}{y\left( r \right)} = 4\pi \kappa_{M} \sqrt {BEt} \frac{{\left( {\tau^{2} - 1} \right)^{\frac{1}{2}} }}{{{\text{arctanh}}\left( {1 - \tau^{ - 2} } \right)^{\frac{1}{2}} }},$$

where $K_{0} \left( x \right)\sim \log \frac{x}{2} \left( {x \ll 0} \right)$ is used. For the pressurized case ($\tau \gg 1$), Eq. (3) is approximated as

4 $$k_{as} \sim \frac{\pi Pf}{{\kappa_{M} \log \frac{{Pf\sqrt {3\left( {1 - \nu^{2} } \right)} }}{{Et^{2} \kappa_{M}^{2} }}}} ,$$

where *f* is the deformation sensitivity as a function of *κ*_M_ and Gaussian curvature *κ*_*G*_ (Eq. 16). Surface geometry of the cell is characterized by the index *f*, i.e., *f* ranges from 3/4 (cylinder geometry) to 1 (sphere geometry) (see also refs. 28, 29, 30). Based on the geometrical parameters of the plant cell in Fig. 3 (Table 1), the sensitivity of the mechanical and geometrical parameters was evaluated using Eqs. (3) and (4). As shown in Fig. 4B, *k*_*as*_ increased as *E*﻿ and *P*﻿ increased, meaning that stiffness depends on mechanical parameters associated with the cell wall. By contrast, the effects of cell wall thickness *t* and curvature *κ*_M_ ﻿were not proportional to *k*_*as*_ because their contributions in Eq. (4) were nonlinear (Fig. 4C).

**Table 1:** Geometrical parameters of the onion epidermal cell in Fig. 3.

Parameters	Meaning	Before LP	After LP
${L}_{a}$[$\upmu$m]	Length along long-axis	$70.5$	$66.5$
${L}_{b}$[$\upmu$m]	Length along short-axis	$27$	$23.5$
$w$[$\upmu$m]	Bulge height of the cell surface	$3.5$	$0.66$
$\kappa_{a} \left( { = \frac{2w}{{L_{a}^{2} + w^{2} }}} \right)$[${\upmu }$m^−1^]	Axial curvature (Curvature along long-axis)	$1.40\times {10}^{-3}$	$2.98\times {10}^{-4}$
$\kappa_{h} \left( { = \frac{2w}{{L_{b}^{2} + w^{2} }}} \right)$[${\upmu }$m^−1^]	Hoop curvature (Curvature along short-axis)	$9.44\times {10}^{-3}$	$2.39\times {10}^{-3}$
$\kappa_{M} \left( { = (\kappa_{a} + \kappa_{h} } \right)/2$[${\upmu }$m^−1^]	Mean curvature	$5.42\times {10}^{-3}$	$1.34\times {10}^{-3}$
$\kappa_{G} \left( { = \kappa_{a} \cdot \kappa_{h} } \right)$[${\upmu }$m^−2^]	Gaussian curvature	$1.32\times {10}^{-5}$	$7.12\times {10}^{-7}$
$f$	Deformation sensitivity	0.818	0.809

### FEM simulations to verify the formulation

**Figure 5:**
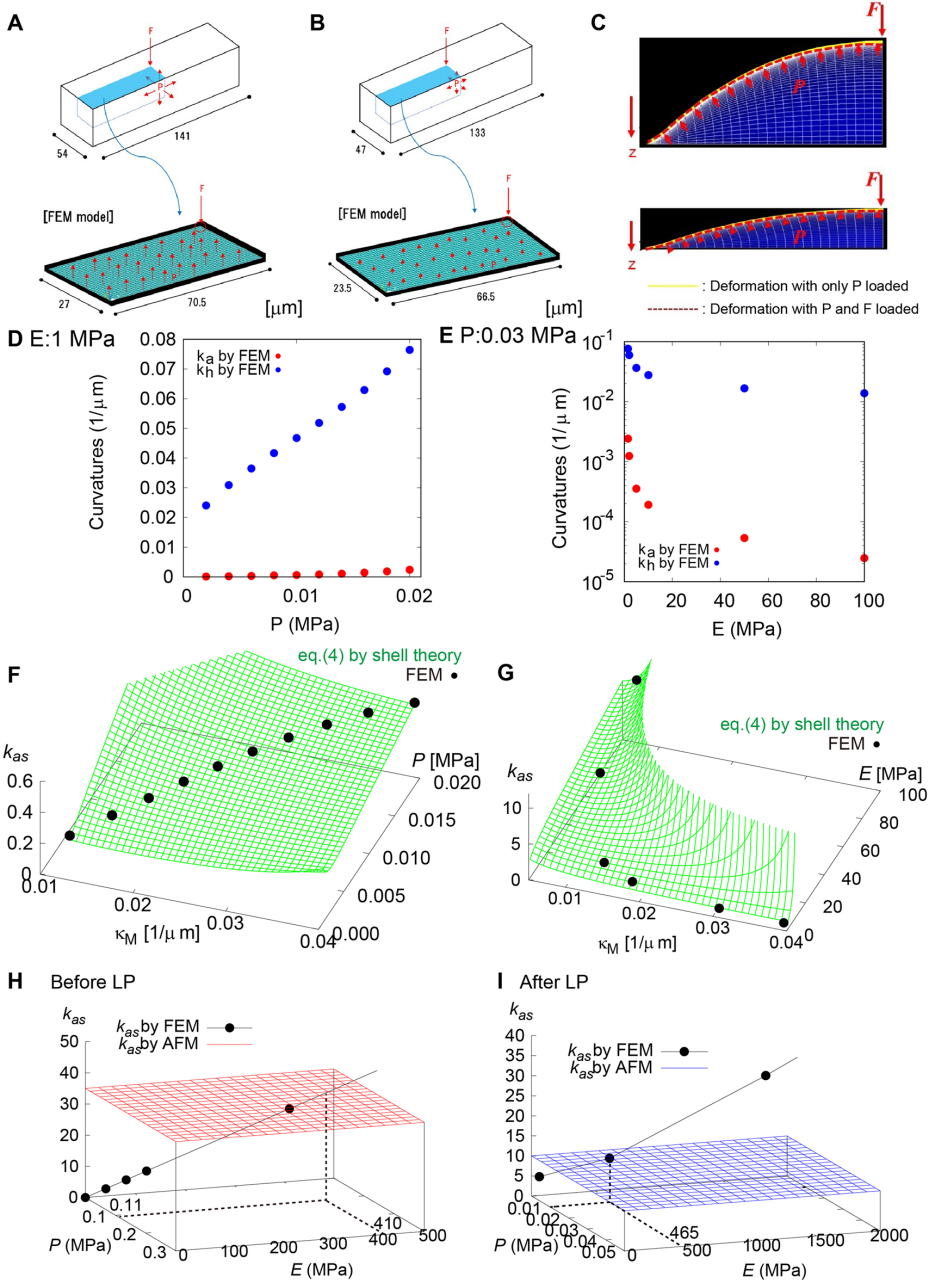
Verification of our theoretical results based on FEM simulation. (**A**, **B**) FEM model based on the actual cell surface geometry before (**A**) and after (**B**) LP. (**C**) Cell surface deformation before (top) and after (bottom) LP. Yellow and red lines are deformations with *P* and with *P* and *F*, respectively. (**D**, **E**) Axial curvature *κ*_*a*_ and hoop curvature *κ*_*h*_ calculated as a function of turgor pressure *P* when *E* = 1.0 MPa (**D**) and as a function of Young’s modulus *E* when *P* = 0.03 MPa (**E**). (**F**, **G**) Apparent stiffness *k*_*as*_ before LP calculated as a function of *κ*_M_ and *P* when *E* = 1.0 MPa (**F**) and as a function of mean curvature *κ*_M_ and *E* when *P* = 0.03 MPa (**G**). Black dots calculated by the FEM simulation are on green surfaces calculated using Eq. (4). (**H**, **I**) Estimation of *E* and *P* using *k*_*as*_ measured before (**H**) and after (**I**) LP. Black dots calculated by the FEM simulation are interpolated using Eq. (4) (black line). Dots on the red and blue planes in (**H**) and (**I**) indicate *E* and *P*, respectively, in agreement with *k*_*as*_ quantified using the AFM measurement.

The reliability of Eq. (4) was verified by the FEM simulation based on the geometrical parameters shown in Fig. 3. To represent a typical plant cell, we prepared a hollow rectangular box with 54 µm width, 54 µm height, and 141 µm length for the cell before LP and with 47 ﻿µm width, 47 µm height, and 133 µm length for the cell after LP (Fig. 5A,B). Cell wall thickness was assumed to be 1.5 µm based on similar values in the literature^9^. Taking geometrical symmetry into consideration, we focused on a 1/4 model of the cell surface and applied a concentrated force on the edge, corresponding to the center of the cell surface.

Deformation of the cell surface with and without the applied force *F* was confirmed as shown in Fig. 5C. The surface geometry before and after LP indicated that cell curvature depends strongly on *P*. Figure 5D shows the *P* dependence of axial curvature *κ*_*a*_ and hoop curvature *κ*_*h*_ (see Fig. 4A), confirming that the cell surface swelled with increasing turgor pressure in the simulation range of *P* (0–0.02 MPa). By contrast, *κ*_*a*_ and *κ*_*h*_ decreased with increasing *E* (Fig. 5E). However, the change was not so drastic in the simulation range of *E* (1 ~ 100 MPa) compared with that of *P*. Although the simulations in Fig. 5D,E were performed under the condition without applied force *F*, this result implies that indentation with *F* depends strongly on *P* rather than *E*, as suggested in Eq. (4).

Apparent stiffness *k*_*as*_ in the FEM simulation was calculated from the indentation caused by loading *F*. Figure 5G,F show the dependency of *κ*_M_ and *E* and that of *κ*_M_ and *P*, respectively. It is worth noting that *k*_*as*_ in the FEM simulation (black points) is on the curved surface calculated by Eq. (4). The good agreement between the theory and simulation supports the reliability of the elastic shell theory. Namely, Eq. (4) has the generality to describe deformation of a rectangular plant cell by external force. When the equation was verified by the FEM simulation, *E* and *P* were determined independently from *k*_*as*_ and *κ*_M_ measured by the AFM experiment.

### Estimation of *E* and *P* based on force curve measurements using AFM

The force–indentation curves of the onion cell before and after LP are shown in Fig. 3F. From this data, even with the very small indentation of ~ 10 nm,﻿ *k*_*as*_ decreased to 1.5 (N/m) after LP from 2.5 (N/m) before LP. If the cantilever only senses the cell wall elasticity, *k*_*as*_ before and after LP should be almost the same. Therefore, this difference in *k*_*as*_ indicates that the indentation process is affected by *P* and *κ*_M_ in Eq. (4), even in a nano-scale indentation.

As shown in Fig. 3G, *k*_as_ increased as indentation increased with the applied force. Although indentation with a force larger than 10 µN was not measured because of the detection limits of our AFM system, *k*_*as*_ converged to a value around 35 (N/m) before LP and to 10 (N/m) after LP. We then performed a combination search to find the best fitted parameters *E* and *P* by FEM simulation satisfying the bulge height *w* of 3.5 µm before LP and 0.66 µm after LP (Fig. 5H,I respectively). *E* and *P* before LP with *k*_*as*_ ~35 N/m were estimated to be ﻿410 and 0.11 MPa, respectively, while those after LP with *k*_*as*_ ~ 10 N/m were E ≈465 and ≈0.01 MPa, respectively. The estimated *P* was in the plausible order of 0.1 MPa reported previously^1,3,16^, which depends on cell condition. By contrast, *E* of around 450 MPa was much larger than estimations given in the literatures from AFM measurement with the Hertz model, which are around the order of 1 MPa^4,9^.

## Discussion

Comparison of force–indentation data for agarose gel and for the onion epidermal cell suggested that the Hertz model with the assumption of an elastic semi-infinite solid is unsuitable for interpreting the apparent stiffness of plant cells, *k*_as_. This means that *k*_*as*_ detected by AFM is dependent on more than simply the Young’s modulus of the cell wall *E*. Theoretical analysis based on the elastic shell theory indicates that the major contribution to *k*_*as*_ is turgor pressure *P*. This fact was confirmed experimentally from AFM measurements before and after LP, in which *k*_*as*_ depended on *P*. In almost all previous literatures regarding AFM detection of plant cells, the stiffness of the plant cell has been estimated using *E* based on the Hertz model. We propose that the estimated value of *E* must reflect not only the cell wall elasticity but also the turgor pressure.

Our formulation based on the elastic shell theory revealed that the contributions of *E* and *P* to *k*_*as*_ are strongly affected by three geometrical parameters of the cell: surface mean curvature *κ*_M_, deformation sensitivity *f*, and cell wall thickness *t* in equation (4). We showed that the quantitative value of *k*_*as*_ predicted from Eq. (4) is well reproduced by FEM simulation assuming a simple, hollow, rectangular box, in which surface curvatures and cell wall thickness are also in good agreement with those in the theory. This result confirmed that these three geometrical parameters (*t*, *κ*_M_, and *κ*_*G*_) are the minimum requirements for estimating *E* and *P* from *k*_*as*_.

Despite good correspondence between the elastic shell theory and FEM simulations, *E* estimated by the elastic shell theory (~ 450 MPa) was larger than *E* estimated by the Hertz theory (~ 1 MPa), not only in our estimation presented in Fig. 2D but also in AFM measurements from the literature^4,9^. One possible reason might be the anisotropic elasticity of the cell wall. The cell wall is organized into distinct cellulose-containing and middle lamella layers, in which callous fibers are arranged in the plane direction. The analysis based on the elastic shell theory estimates *E* of the in-plane direction of the cell wall. By contrast, estimation based on the Hertz theory strongly reflects *E* in the out-of-plane direction. The composite structure of the cell wall implies that the in-plane elasticity is higher than the out-of-plane elasticity.

The in-plane elasticity of the onion epidermal cell wall has been measured previously using a microelectromechanical system (MEMS) tensile testing device^33^. In this experiment, epidermal peels were cut to 15 × 5 µm using a focused ion beam and fixed in a gap between movable and fixed beams in the MEMS, which were connected to a piezoelectric actuator and a force sensor, respectively, under atmospheric conditions. *E* measured from the shift of the actuator and force detected by the sensor was 3.7 GPa. As *E* is probably affected by water swelling of the sample, it seems reliable that *E* in our estimation (~ 450 MPa) performed in water is a little smaller than *E* estimated by the MEMS. Our method is more convenient and suitable for estimating in-plane elasticity of the cell wall compared with the method utilizing the MEMS, which requires skillful manipulation of the cell wall sample.

Previously, Beauzamy et al.^9^ investigated the contributions of *P* and *E* from AFM measurements using experiments with osmotic pressure control to modify *P*. They suggested a relationship between *k*_*as*_ and *P* based on elastic shell theory; however, their estimation was performed with the estimated value *E* derived from the Hertz model. By contrast, we employed FEM simulation to estimate *E* and to verify the relationship between *k*_*as*_ and *P* quantitatively. Our results suggest that the actual contribution to cell wall stiffness may be the combination of the in-plane elasticity and cell wall tension, which can be modified flexibly by a slight adjustment of turgor pressure.

Our estimation of turgor pressure (~ 0.1 MPa) was smaller than that reported previously (0.3 ~ 1.0 MPa)^1,3,16^. The detection limitation of our AFM system prevented measurement until *k*_*as*_ was saturated and became constant, with perfect contribution to the in-plane elasticity of the cell wall. It is possible that *k*_*as*_ was evaluated to be smaller than the true value. As *P* is almost proportional to *k*_*as*_, a larger measurement of ﻿*k*_*as*_ might result in larger *P*. Another possibility is the simple assumption of cell structure in the elastic shell theory and FEM simulation. The cell wall is not a simple plane but instead a curved plane containing a nano-microstructure of cellulose fibers reinforced by the cytoskeleton. In addition, the plant cell is not hollow but filled with cytoplasm and many cell components. These factors may upset the estimation of *P*. Our theoretical formulation implies that the shell structure of plant cells has mechanical advantages. The cylindrical surface geometry of onion epidermal cells stabilizes their structure. Stiffness is flexibly modified by a slight adjustment of turgor pressure in the order of 0.1 MPa. Such structural stabilization would apply not only to a single cell but also to tissues as complexes of cells.

Since onion epidermal cells are originally differentiated from cells in the stem, we expect that they maintain a convenient shape for supporting this cylindrical structure. The structure has advantages for mechanically supporting leaf weight. In this case, securing sufficient light is an essential factor for changing cell shape. In addition, plant cells may deform into a shape with a different ratio between volume and surface area to effectively transport internal and external substrates. This consideration indicates that the mechanical and geometrical properties of plant cell structure are a function of plant physiological state, such as photosynthesis and substrate transport. Therefore, the next important goal might be to identify correlations between stiffness, material, mechanics, geometry, and physiology in the cells of many different organs and/or species, thus opening a new research framework for enhancing interdisciplinary collaboration between plant science, physics, and mechanical engineering.

## Methods

### AFM measurement

Epidermal tissue from scale leaf of the yellow onion (*Allium cepa* L.) was prepared for the laser perforation and the AFM measurements. The scale leaf was cut into 10 mm squares using a razor. The single-layer epidermis was peeled off from the cut leaf using tweezers. The epidermis was placed on a glass-bottom dish, and mounted on an inverted microscope (Olympus, IX71), as shown in Fig. 1A. We measured the cuticle side of the epidermis, which is upper side of the placed sample. The AFM system (JPK Instruments, Nanowizard 4) was attached to the microscope stage. The AFM cantilever probes used to evaluate tip radius dependence (Fig. 2) were TL-NCH (Nanosensors) with tip radii of 0.4, 5, and 10 µm*.* The AFM cantilever probes with laser perforation (Fig. 3) were NCHR (NanoWorld) with a 16 nm tip radius for topography imaging (Fig. 3B,C) and SD-Sphere-NCH-S (Nanosensors) with a 400 nm tip radius for force–indentation curve measurements (Fig. 3F,G). The spring constant* k* of all cantilevers was 40 N/m. The space between the cantilever and the sample was filled with water. The detection positions of interest were chosen by adjusting the cantilever position at the center of the cell. All methods were carried out in accordance with relevant guidelines.

### Pulsed-laser perforation method

Laser pulses from a regeneratively amplified Ti:Sapphire femtosecond laser (800 ± 5 nm, 100 fs, < 1 mJ/pulse, 32 Hz) (Spectra-Physics, Solstice Ace) were introduced to the microscope through a × 20 objective lens (Olympus, UMPlan FL, NA = 0.46). The pulse was focused on the cell wall. The laser focal position was adjusted to the center of the cell along the short axis and at 50 µm away from the AFM probe position along the long axis, so that the perforation would not interfere with detection. Perforation was controlled by a mechanical shutter (Sigma Koki, Σ-65GR) with a gate time of 1/32 s. The laser pulse energy was tuned from 50 to 200 nJ/pulse using a half-wave plate, a polarizer, and a neutral density (ND) filter in the optical path. AFM measurements were performed before and after LP on the cell wall as shown in Fig. 1B. Deformation of the cell wall was monitored using a CMOS camera (WRAYMER, FLOYD-100).

### Estimation of Young’s modulus using the Hertz model

The force–indentation curve represents the relationship between the force *F* applied to the sample surface by the AFM probe and the depth *d* of the surface indentation created by the applied force. In the AFM measurement, *F* is obtained from the tip displacement﻿ *D*_*P*_ of the cantilever resulting from deformation and the spring constant *k* of the cantilever determined by Hooke’s law:

5 $$F = kD_{p} .$$

The indentation depth *d* is estimated by subtracting *D*_*p*_ from the whole displacement Δ*z* of the cantilever:

6 $$d = {\Delta z} - D_{p} { }{\text{.}}$$

In Hertz’s contact theory for the relationship between a flat plate and a protrusion having curvature radius *R*_tip_, *F* and *d* are related as

7 $$F = \frac{4}{3}\frac{E}{{1 - \nu^{2} }}\sqrt {R_{{{\text{tip}}}} } d^{\frac{3}{2}} { }{\text{.}}$$

Young’s modulus *E* was estimated by fitting the experimental data using a least-squares method. In this fitting, Poisson’s ratio *ν* was assumed to be 0.5, which is the theoretical maximum.

### Definition of mechanical and geometrical parameters for elastic shell theory

The Eq. (1) contains functions of the bending modulus *B*, the mean curvature *κ*_M_ and the uniform stress on the shell surface σ∞ in Eq. (1), which are denoted as

8 $$B = \frac{{Et^{3} }}{{12\left( {1 - \nu^{2} } \right)}},$$

9 $$\kappa_{M} = \frac{{\kappa_{a} + \kappa_{h} }}{2}\,{\text{and}}$$

10 $$\sigma_{\infty } = \frac{Pf}{{2\kappa_{M} }},$$

where *κ*_a_ and *κ*_h_ are the axial and hoop curvatures, corresponding to curvatures along long- and short- axis, respectively. The Eq. (2) is derived from the Hankel transformation of order 0 for Eq. (1), with

11 $$\overline{y}\left( s \right) = \int_{0}^{\infty } {y\left( r \right)J_{0} \left( {kr} \right)rdr} ,$$

where *J*_*0*_ is the Bessel function of order 0 (second kind), written as

12 $$Bs^{4} \overline{y} - \sigma_{\infty } s^{2} \overline{y} + Et\kappa_{M}^{2} \overline{y} = - \frac{F}{2\pi }.$$

The indices ﻿λ± in Eq. (2) are the solutions of an index *s* in the denominator of the following equation,

13 $$\overline{y} = - \frac{F}{{2\pi \left( {Bs^{4} - \sigma_{\infty } s^{2} + Et\kappa_{M}^{2} } \right)}}.$$

The bending scale﻿ *l*_*b*_, the dimensionless pressure τ in Eq. (2) are denoted as

14 $$l_{b} = \left( {\frac{B}{{Et\kappa_{M}^{2} }}} \right)^{\frac{1}{4}} ,$$

15 $$\tau = \frac{{Pf\sqrt {3\left( {1 - \nu^{2} } \right)} }}{{2Et^{2} \kappa_{M}^{2} }}.$$

The deformation sensitivity *f* in Eq. (15) is denoted as

16 $$f = {{\left( {2 + \frac{{\sqrt {1 - \kappa_{G} /\kappa_{M}^{2} } - 1}}{{\kappa_{G} /\kappa_{M}^{2} }}} \right)} \mathord{\left/ {\vphantom {{\left( {2 + \frac{{\sqrt {1 - \kappa_{G} /\kappa_{M}^{2} } - 1}}{{\kappa_{G} /\kappa_{M}^{2} }}} \right)} {\left( {\sqrt { 1 - \kappa_{G} /\kappa_{M}^{2} } + 1} \right)}}} \right. \kern-\nulldelimiterspace} {\left( {\sqrt { 1 - \kappa_{G} /\kappa_{M}^{2} } + 1} \right)}},$$

where Gaussian curvature﻿ *κ*_*G*_ is the product of the axial and hoop curvatures: $\kappa_{G} = \kappa_{a} \cdot \kappa_{h}$. The parameters used in the present calculation are summarized in Table 1.

### FEM simulation

The simulation was conducted using the general FEM software ANSYS (ver. 2019 R3). To simulate the mechanical behaviors of the cell wall under turgor pressure and applied force, SHELL181 was selected as the element of the numerical model. SHELL181 is a shell element with four nodes, each of which has three translational and three rotational degrees of freedom (DOFs). This element can be used to investigate the effect of the thickness of cell wall structures, as the thickness can be defined manually, ranging from cases of thin shells to thick shells. The numerical model illustrated in Figs. 5A,B was divided into 100 parts in the *x* direction and 15 parts in the *y* direction. Therefore, the FEM model was constructed using 1500 SHELL181 elements.

To increase the calculation efficiency, one-fourth of the top surface was utilized to analyze cell wall structure. Boundary conditions and loads were defined according to the symmetricity. For nodes at the peripheral boundaries (left and lower edges of the FEM model in Fig. 5A,B), all the DOFs were restricted. For nodes at mirror surfaces (right and upper edges of the FEM model in Fig. 5A,B), the DOFs were partially restricted according to the symmetricity. One-fourth of the applied force from the AFM probe was introduced as nodal force onto the top surface. The turgor pressure was simulated as a uniformly distributed load that maintained direction along the normal vectors of the surface during deformation.

The numerical calculation included two stages. In stage 1, only the turgor pressure was defined in the model; in stage 2, the applied force was introduced as well. The calculation was conducted with consideration of geometrical nonlinearity using the Newton–Raphson method. To reach computational convergence efficiently, the loads in stages 1 and 2 were introduced into the model in 20 steps and 50 steps, respectively.

## Data Availability

The datasets used and/or analysed during the current study available from the corresponding author on reasonable request.
